# Spatially Controlled All-Optical Switching of Liquid-Crystal-Empowered Metasurfaces

**DOI:** 10.1021/acsphotonics.4c02029

**Published:** 2025-01-21

**Authors:** Maximilian Beddoe, Sarah L. Walden, Slobodan Miljevic, Dmitry Pidgayko, Chengjun Zou, Alexander E. Minovich, Angela Barreda, Thomas Pertsch, Isabelle Staude

**Affiliations:** † Institute of Solid State Physics, Friedrich Schiller University Jena, 07743 Jena, Germany; ‡ Institute of Applied Physics, Abbe Center of Photonics, Friedrich Schiller University Jena, 07745 Jena, Germany; ¶ School of Environment and Science, Griffith University, 170 Kessels Road, Nathan 4111, Australia; § Institute of Microelectronics, Chinese Academy of Sciences, Beitucheng west road 3, Beijing 100029, China; ∥ Group of Displays and Photonics Applications, Carlos III University of Madrid, Avda. de la Universidad, 30, Leganés, 28911 Madrid, Spain; ⊥ Fraunhofer Institute for Applied Optics and Precision Engineering, Albert-Einstein-Str. 7, 07745 Jena, Germany

**Keywords:** tunable metasurfaces, active tuning, liquid crystals, photoalignment, Mie-resonances, all-dielectric nanophotonics

## Abstract

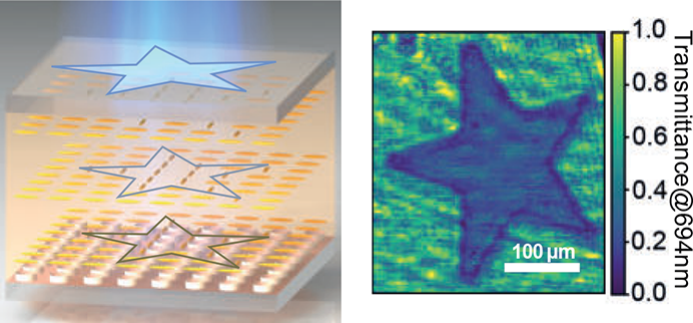

Embedding metasurfaces in liquid crystal (LC) cells is a promising technique for realizing tunable optical functionalities. Here, we demonstrate spatially controlled all-optical switching of the optical response of a homogeneous silicon nanocylinder metasurface featuring various Mie-type resonances in the spectral range between 670 and 720 nm integrated in a nematic LC cell. The initial alignment of the LC molecules is controlled by photoalignment layers, where the alignment direction is defined by homogeneous exposure with linearly polarized light at a 450 nm wavelength. Exposure of the photoalignment layer with the same light, whose polarization is rotated by 90°, induces a local change in the direction of the LC alignment and modulates the optical response of the metasurface. The resulting spatially dependent optical properties of the metasurface system are characterized by hyperspectral imaging. The described technique allows the nonvolatile creation of complex spatio-spectral response functions with a spatial resolution of 20 μm. Moreover, we demonstrate that the response of the LC-integrated metasurface can be switched multiple times by subsequent exposures with alternating orthogonal polarizations. Finally, we show that the images can be temporarily erased by heating the sample above the critical LC transition temperature, where the LC transitions to its isotropic phase. The demonstrated approach represents the controlling-light-by-light concept, an alternative to electro-optical or electromechanical control methods, which require complicated electronic architectures for spatially resolved modulation. Our results hold significant potential for applications such as next-generation displays or spatial light modulators that require spatial control of a tunable, tailored optical response.

## Introduction

Optical metasurfaces consist of a 2D-tailored arrangement of subwavelength scatterers, often called meta-atoms. Over the past decade, optical metasurfaces have revolutionized the field of nanophotonics.^1−3^ Low-loss, all-dielectric metasurfaces can be fabricated with well-established lithographic techniques^4^ and exhibit high interaction efficiency with light at the nanoscale.^5,6^ To date, numerous efficient, flat optical devices have been reported from all-dielectric metasurfaces, including holograms,^7,8^ images,^9−11^ metalenses,^12−14^ wavefront shapers,^15^ and polarization converters.^16^ However, most metasurfaces and metadevices realized thus far are static with their optical functionality fixed upon fabrication. With many potential applications of metasurfaces profiting from, or even relying on, the possibility of altering their optical response postfabrication in a controlled and reversible fashion, dynamically tunable and switchable metasurfaces have become an active area of research, and a range of different tuning approaches have been demonstrated.^2,17−19^ A common strategy to introduce tunability into optical metasurfaces is to modify the refractive index of the medium surrounding the meta-atoms.^20−22^ One way to achieve this is to integrate dielectric metasurfaces into nematic liquid crystal (LC) cells.^23−29^ Nematic LCs typically exhibit two phases. In the nematic phase, the LC molecules self-align with their long axes roughly parallel, forming an anisotropic optical material described by a uniaxial refractive index tensor, with the anisotropy axis pointing along the alignment direction. At elevated temperatures, the LCs undergo a phase transition from the nematic phase to the isotropic phase, in which the molecules are randomly oriented,^30^ causing the LC refractive index to become isotropic.^31^ To our knowledge, Sautter et al. were the first to report thermally tuned transmission spectra of Mie-resonant dielectric metasurfaces based on this effect.^29^ In addition to temperature tuning, the LC alignment direction can also be controlled by the application of a voltage or magnetic field. This, in turn, allows for electric^23,28,32^ or magnetic^25,26^ tuning over the optical response of a metasurface integrated into an LC cell. Zou et al. demonstrated that combinations of two different stimuli, in their case voltage and temperature, could be exploited simultaneously for heightened functionality.^24^

Exciting prospects for LC tunable metasurfaces, including spatiotemporal control, could be achieved by using light as a stimulus.^33^ There are various ways in which light may influence the optical properties of the LCs. Intense optical fields may cause a direct realignment of the LC molecules within their oscillating electric field or induce heating leading to localized phase transitions.^34^ Dynamic reorientation of LCs can further be produced from lower intensity light exposure mediated by additional light-responsive components such as photoalignment materials or dyes.^35−37^ In the context of LC tunable metasurfaces, however, such photoalignment materials were so far only used to define a global LC prealignment direction for the cells,^23,24,32^ not in order to implement light-induced dynamic control of the LC metasurface response. Many potential applications, such as displays or spatial light modulators, require spatially variant tunable metasurface architectures. In a simple approach toward that goal, polarization-dependent and spatially variant metasurfaces were combined with separate LC cells, imparting a global phase shift on the incident light to realize switchable holograms^38^ or color images.^11^ The integration of all-dielectric metasurface-based beam deflectors into nematic LC cells was also demonstrated to enable a dynamic redistribution of light into different diffraction orders.^39^ In order to flexibly modify the optical properties of a metasurface as a function of the in-plane position, as required for reconfigurable images or reprogrammable diffractive optical components, spatial control over the application of the stimulus is required. Up to now, this has been achieved by spatially varying the applied voltage using one-dimensional arrays of stripe-shaped electrodes.^40−44^ However, the fabrication of such devices is difficult, and sophisticated control electronics become necessary as the number of electrodes increases. Moreover, the extension of the concept to 2D-pixel arrays, as needed for many of the envisioned applications, presents a challenge. Similar arguments apply for position-selective tuning using local resistive heaters.

Structured light offers intriguing new opportunities for the flexible spatially controlled switching of LC-integrated metasurfaces. Instead of relying on complex control schemes and integration, almost arbitrary two-dimensional intensity distributions can be readily generated on the LC-integrated metasurface using common spatial light modulators, such as digital mirror devices (DMDs).^45^ DMDs have previously been used to generate LC-based patterns,^45,46^ waveguides,^47^ Q-plates,^48,49^ and vortex beams,^50^ but not for the spatially resolved tuning of LC-integrated metasurfaces. Theoretically, the resolution of a generated light pattern is determined by the wavelength and the numerical aperture of the projection system (objective) and can reach subwavelength values within the metasurface operating band if the controlling light is significantly blue-shifted. However, in practice, the feature sizes are limited by the molecular dynamics of the LCs, and the resolution depends on the LC thickness.

Here, we demonstrate spatially controlled, all-optical switching of LC integrated silicon metasurfaces featuring Mie-type resonances. Specifically, we use photoalignment layers for the LCs and illumination with structured, polarized blue light to locally change the in-plane orientation of the LCs within controlled spatial patterns. Using hyperspectral imaging with linearly polarized light, we measure the spatially resolved transmittance before and after exposure, revealing a pronounced spectral shift of the Mie-resonances within the exposed region. We furthermore demonstrate multiple light-induced switching cycles by exposure of the photoalignment material to the blue light of alternating orthogonal linear polarizations. Finally, we show that the obtained spatial pattern can be temporarily erased by heating the sample and inducing a phase transition of the LC into the isotropic phase. Time-resolved transmission measurements are performed to reveal the dynamics of this process.

## Main Principle

**Figure 1:**
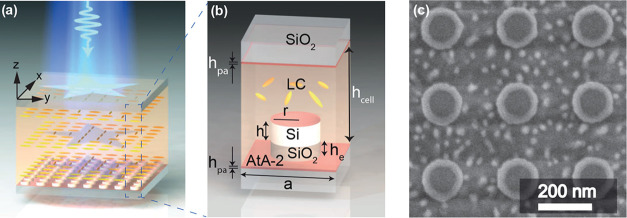
(a) Schematic illustration (not to scale) of a silicon nanocylinder metasurface embedded in an LC cell exhibiting spatially engineered domains (here, star-shaped) of different in-plane alignment directions. (b) Sketch (not to scale) of the metasurface unit cell. (c) Top-view SEM image of the metasurface.

The design principle for achieving spatially controlled all-optical switching of the optical response of a metasurface integrated into a nematic LC cell is depicted in Figure 1a. We focus on the case of a dielectric metasurface comprised of a periodic array of Si nanocylinders on SiO_2_ substrate featuring electric and magnetic dipole resonances^24^ around 700 nm. The metasurface is coated with a thin layer of the photoalignment material AtA-2, which provides sufficient anchoring energy to induce the LC alignment throughout the cell volume.^51^ AtA-2 consists of linear azobenzene photoswitches that selectively absorb light polarized along the axis of the molecule. Upon absorption, azobenzene molecules undergo isomerization and gradually reorientate to be perpendicular to the polarization direction of incident blue light at a low exposure dose.^23,52,53^ This process results in a local rotation of the LC director within the cell. We initially set the LC orientation over the entirety of the LC cell. Then, a particular region (star in Figure 1a) is exposed to spatially structured, polarized light with the wavelength of 450 nm to induce a 90° reorientation of the photoalignment layer which is followed by the same in-plane rotation of the LC. This reorientation causes a local change of the anisotropic refractive index surrounding the nanocylinders, consequently modifying the optical response of the metasurface in the exposed region. Azobenzene is well-known to be highly photostable. Trans and cis states can be switched reversibly with low-power light for 10^5^ and 10^6^ cycles before chemical fatigue.^54^ Between irradiation cycles, azobenzene photoalignment materials have been shown to be extremely stable even under exposure to extreme conditions (100 °C for 8 h)^55^; however, their performance is known to decrease in high humidity environments.^56^ The metasurface was fabricated using electron beam lithography (EBL) followed by reactive ion etching (see ref (24) for details on the fabrication procedure). The resulting nanocyclinders have a radius of *r* = 100 nm, a height of *h* = 105 nm, an SiO_2_ pedestal resulting from slight overetching with a height of *h*_e_ = 38 nm and a period of *a* = 360 nm (see Figure 1b for a sketch of the unit cell). The metasurface and a SiO_2_ slide, serving as the upper window of the LC cell, were coated with a thin layer of AtA-2 with a thickness *h*_pa_ of approximately 10 nm by spin-coating. Prior to assembling the LC cell, the AtA-2 layers were exposed to *x*-polarized 450–455 nm LED light (12.3 W/cm^2^) for 90 s in order to set the prealignment direction of the LCs along the *y*-direction.^23,32^ A scanning electron microscopy (SEM) image of the sample is shown in Figure 1c.

Once assembled, the height of the LC cell was measured as *h*_cell_ ≈ 10 μm. Finally, the nematic LC E7 (Merck Licrystal) was injected into the cell (see the Supporting Information for details on cell construction). E7 has a critical temperature of 58 °C, an ordinary refractive index *n*_o_ = 1.53, and an extraordinary refractive index *n*_e_ = 1.75.^31^ To reorient the LC molecules in the desired region, the LC cell was exposed with 450 nm wavelength, continuous-wave (CW) *y*-polarized laser light at an intensity of 4.1 W/cm^2^ for 4.5 min. The exposure was performed in the isotropic state as exposing the LC cell in the nematic state would result in a twisted nematic cell.^57^ Spatial control of the LC reorientation was realized using a DMD to spatially structure the laser light (see Supporting Information for setup details). The spatially resolved metasurface transmittance was then recorded by hyperspectral imaging using a supercontinuum laser (NKT Photonics, SuperK EXTREME) with a monochromator (SuperK Select) as the source and a Zelux 1.6 MP Monochrome CMOS Camera as the detector and compared with corresponding simulated spectra (see Supporting Information for setup details).

## Results

**Figure 2:**
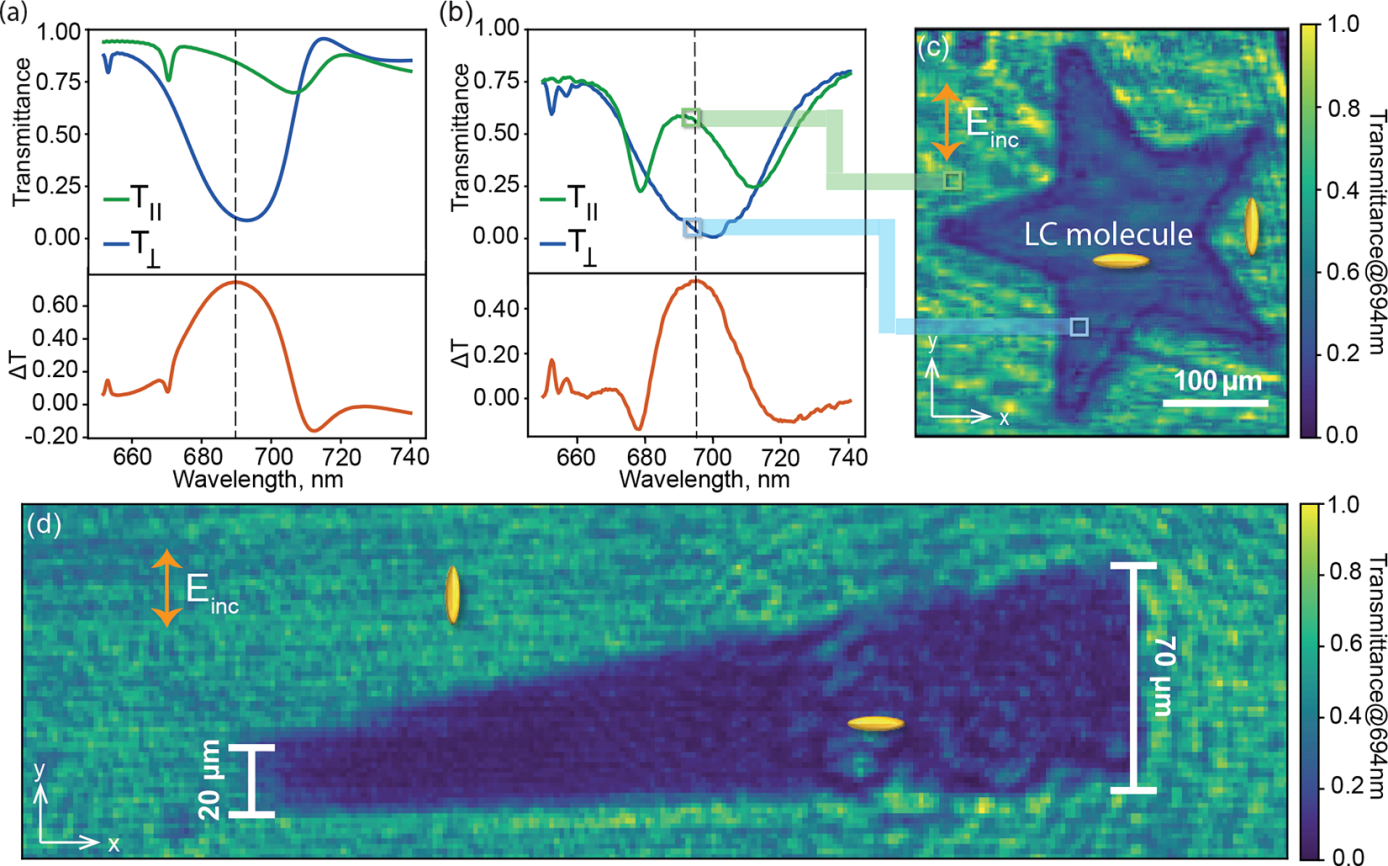
(a) Simulated and (b) experimentally measured transmittance spectra *T*_∥_ and *T*_⊥_ for *y*-polarized incident light and *y*- and *x*-oriented LCs, respectively. The transmittance difference Δ*T* = *T*_∥_ – *T*_⊥_ is also shown for both simulated and experimental spectra. (c) Spatially resolved transmittance at 694 nm wavelength for a DMD exposure in the shape of a star. (d) Spatially resolved transmittance map for a triangular-shaped exposure at 694 nm to determine the spatial resolution.

Numerically calculated transmittance spectra of the LC-integrated metasurface for incident *y*-polarized light are shown in Figure 2a for both *y*- and *x*-oriented LCs. To emphasize the relative orientation between the polarization direction of the incident light during the measurement with the electric field (*E*_inc_) and the LC alignment direction, we refer to these transmittance spectra as *T*_∥_ and *T*_⊥_, respectively. The simulations were performed using the finite-difference time-domain method implemented in the commercial software Ansys Lumerical. The metasurface was excited by a plane wave propagating along the negative direction of the *z*-axis and linearly polarized along the *y*-axis. We used experimental data for the dispersive optical properties of Si and SiO_2_. The LC was modeled as an anisotropic homogeneous medium with *n*_o_ = 1.53 and *n*_e_ = 1.75, with the anisotropy axis of the LC in the region above the cylinders pointing parallel to the metasurface plane in the specified prealignment direction. In the region between the cylinders, the LCs were additionally tilted in the *z*-direction to effectively account for alignment inaccuracies mediated by the nanostructured and confined topography.^29,58^ A tilt value of 10° yielded the best agreement with the experimental data.

For *T*_∥_, which corresponds to the polarization pointing along the prealignment direction in an experiment, the simulated transmission spectrum shows two shallow resonant features in the spectral range of interest, one at 710 nm and another at 670 nm. As reported by Zou et al.,^24^ the shallow resonance at 710 nm originates from the interplay of an electric dipole (ED) and a magnetic dipole Mie-type mode (MD), which are in partial spectral overlap, bringing them close to the Huygens’ regime of high transparency.^6^ The dip at 670 nm is a dark mode, which becomes accessible for excitation by an external plane wave by the anisotropy of the LC^23,24^ (see Supporting Information for near-field maps). For *T*_⊥_, equivalent to the regions where the LCs were exposed to spatially structured light in the experiment, the response is dominated by a broad resonant feature, again originating from the interplay of the same ED and MD modes. The change in incident polarization changes the effective refractive index of the individual environments seen by the two modes due to their different vectorial near-field distributions, reducing their spectral overlap and resulting in a more pronounced and blue-shifted minimum.^6^

Corresponding experimental transmittance spectra for *y*-polarized incident light measured inside (*T*_⊥_) and outside (*T*_∥_) of the DMD-exposed regions are shown in Figure 2b. While the spectra in both regions show similar features as the numerically calculated spectra, deviations are observed mainly in the widths and depths of the excited resonances when the LCs are orientated parallel to the incident light. These deviations can be explained by uncertainties in the orientation of the LCs near the nanocylinders^58,59^ and by fabrication tolerances.^59^ Overall, these results clearly show that our system supports pronounced light-induced spatially resolved switching of the optical response of resonant metasurfaces. These findings underpin our assumption that polarized blue light reorients the At A-2 layer in the exposed regions, rotating the LC alignment and thereby the anisotropy axis of the refractive index in the vicinity of the meta-atoms.

To determine the wavelength providing the highest contrast between the prealigned and exposed regions, we further calculated the difference spectrum Δ*T* = *T*_∥_ – *T*_⊥_ (see Figure 2a,b bottom). The maximum experimental value of Δ*T* = 0.5 is located around 694 nm and is indicated by the black dashed line in Figure 2a,b.

Next, the spatially resolved transmittance of the LC-integrated metasurface was recorded at 694 nm, exhibiting the expected decrease in transmission in the region of the metasurface that was exposed to blue light (Figure 2c) and revealing the star-shaped pattern produced by the DMD.

While in this first demonstration of the suggested scheme, we have used Mie-resonant metasurfaces with fairly broad resonances and no engineered polarization sensitivity, conceptually our approach gives access to the many degrees of freedom of light that can be controlled by various kinds of metasurfaces, such as spectrally and angularly sensitive optical response, polarization-sensitive response (including complex polarizations), and active architectures for tuning, e.g., light emission or nonlinear responses.^2^ This is in stark contrast to simple, spectrally weakly disperse phase-only tuning of common spatial light modulators. Already, in our work, we could show that the Mie resonances enable the switching contrast of the images to depend strongly on the frequency, with the highest contrast coinciding with the spectral range of the resonances, which can be tailored by the metasurface design. To further underpin this point of resonance tailoring, we present experimentally measured spectra and spatially resolved transmittance data for another metasurface with varied geometrical parameters in the Supporting Information, showing wavelength-dependent contrast reversal.

To estimate the spatial resolution of this method, we changed the DMD-exposed pattern to a triangle with a side length of 70 μm in the *y*-direction. The transmission map at 694 nm is presented in Figure 2d.

While the triangle can be clearly recognized as long as the height exceeds 20 μm, below this size, the triangle cannot be resolved. This indicates that the resolution of this method is around 20 μm. Note that higher resolutions can potentially be achieved with optimized projection optics and a thinner LC cell.

**Figure 3:**
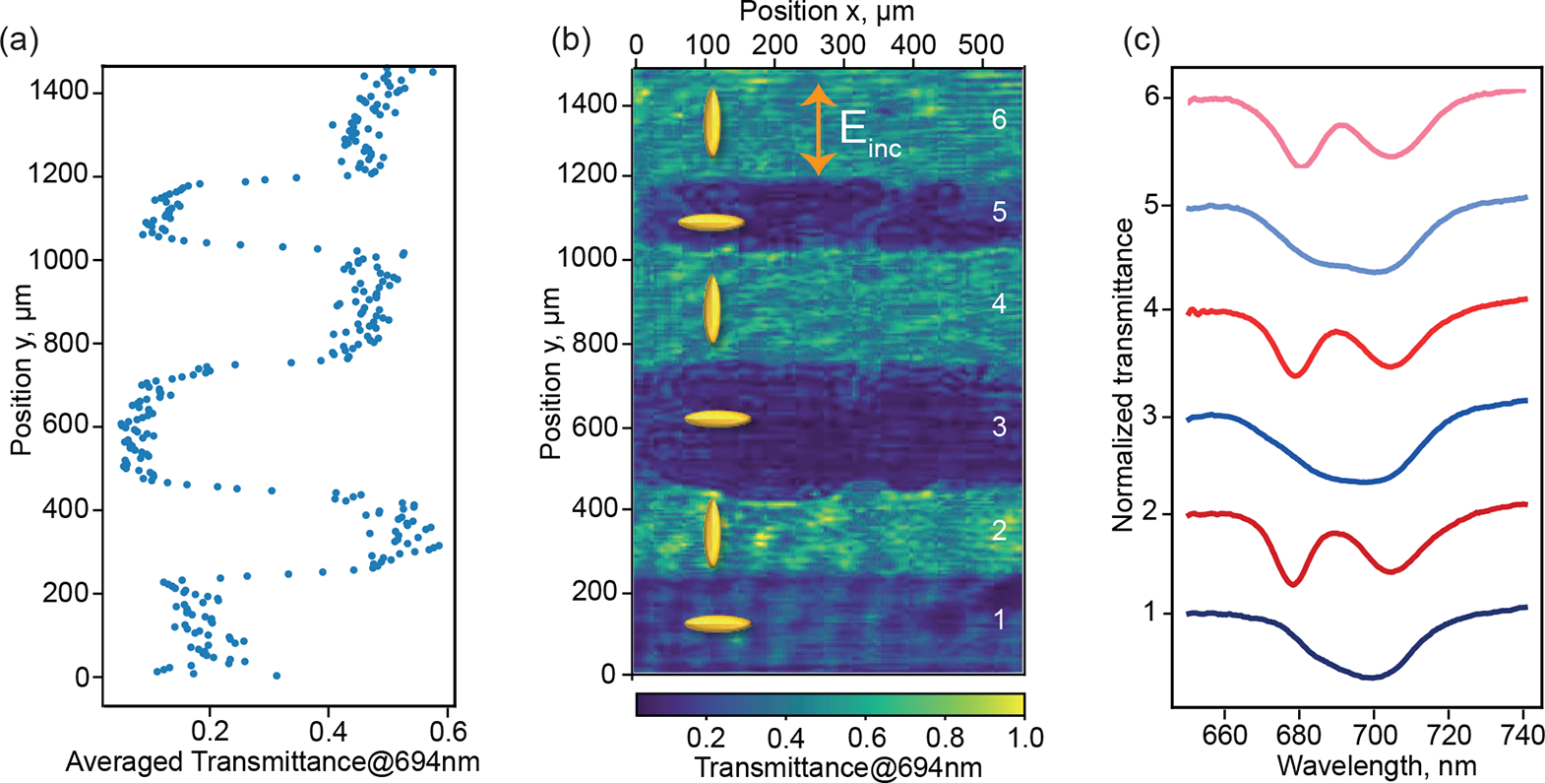
(a) *y*-position-dependent transmission of the LC-integrated metasurface for a 694 nm wavelength and *y*-polarized incident light. The values are averaged over the *x*-coordinate in a line-by-line fashion. (b) Spatially resolved transmission at 694 nm (incident light *y*-polarized). (c) Averaged transmission spectra for different exposure cycles. Subsequent spectra are displaced vertically by one unit to improve the representation.

For many potential applications of reconfigurable metasurface devices, it is important that the system supports multiple switching cycles. In our case, this translates to multiple DMD exposures, where subsequent exposures with different orientations of the linear polarization direction overwrite the alignment conditions established by the previous exposure. Such reconfigurability has already been established for LCs in combination with photoalignment materials in different contexts. For example, Ertman et al.^47^ have erased periodic gratings for waveguides and created new gratings by re-exposing a suitable LC cell. Below, we demonstrate that our system can be exposed multiple times without noticeable deterioration of the optical response of the LC-integrated metasurface in both alignment states. To this end, starting with *y*-polarized blue light exposure (leading to LCs oriented perpendicular to the *y*-polarized incident field), the LC cell was sequentially exposed to rectangular-patterned beams of alternating orthogonal linear polarizations. With each exposure, the length of the rectangle was systematically reduced in the *y*-direction. This resulted in a series of neighboring regions that experienced a different number of exposures. Figure 3a,b presents the 1D- and 2D-spatially resolved transmissions, respectively, for 694 nm wavelength and *y*-polarized incident light after six subsequent DMD exposures. The results clearly showing the expected stripe pattern, highlighting the different transmission intensities in the various regions. The regions with even (*T*_∥_) and odd (*T*_⊥_) numbers of exposures can be clearly distinguished. Note that the initial prealignment was in the *y*-direction (prealigned region not shown in Figure 3b). The spatially averaged transmittance spectra for all six regions are presented in Figure 3c. Spectra for subsequent exposures are displaced vertically by 1 unit for better visibility. As expected, the spectra recorded in all regions with even exposures look similar, as do the regions with an odd number of exposures. This confirms that switching of metasurface resonances using photoalignment of LCs is a reversible process. Thus, this offers important avenues for reconfigurable metadevices. A quantitative comparison between the spectra measured for nominally identical system configurations can be found in the Supporting Information.

**Figure 4:**
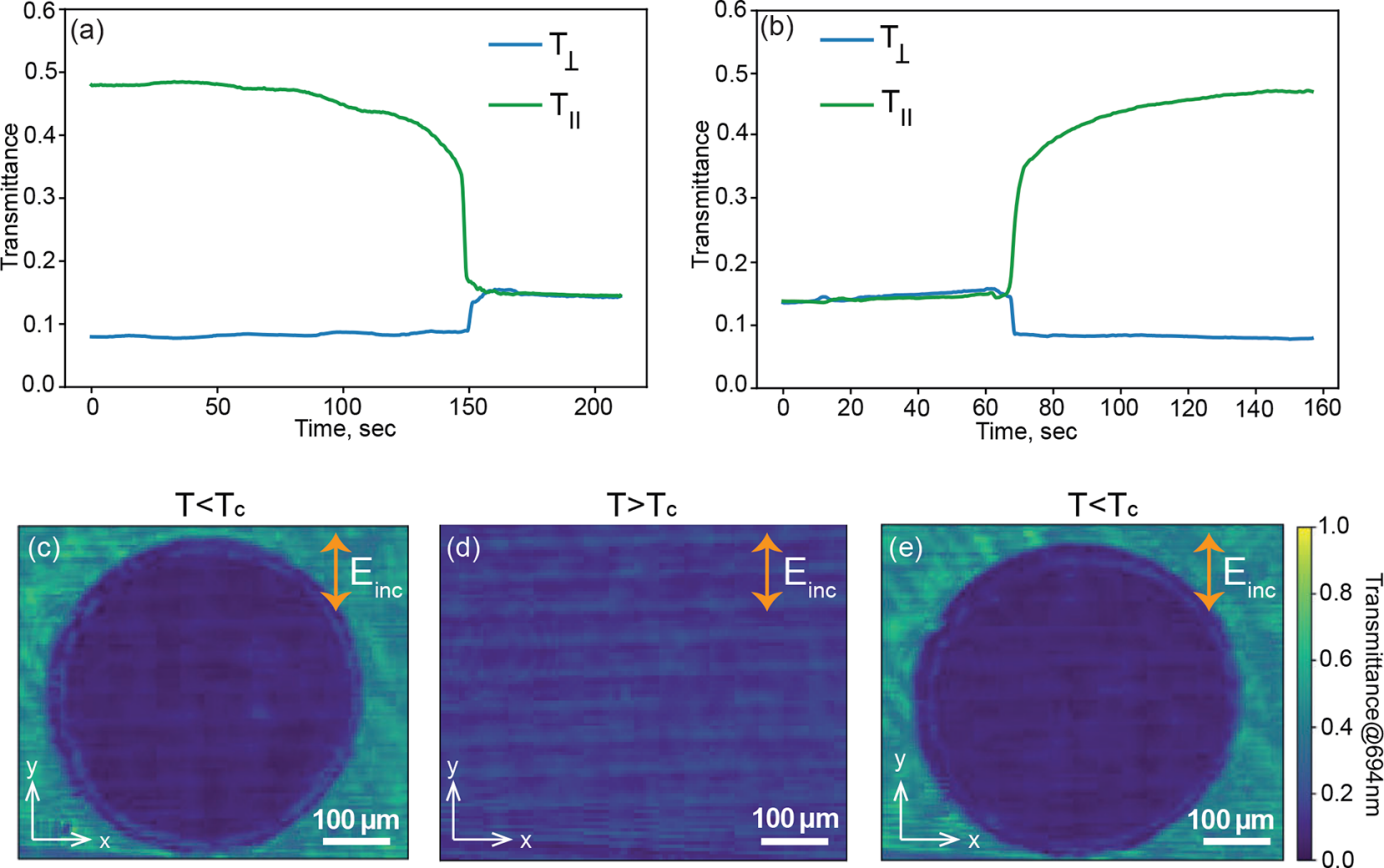
(a, b) Time-resolved transmission of the LC integrated metasurface during (a) heating and (b) cooling, measured both inside (*T*_⊥_) and outside (*T*_∥_) of the DMD-exposed region. (c–e) Spatially resolved transmission (c) before heating, (d) at *T* > *T*_c_, and (e) after cooling. All measurements (a–e) were performed for a 694 nm wavelength and *y*-polarized incident light.

While the demonstrated reconfigurability of the spatially varying metasurface response using DMD exposure already offers a substantial increase in flexibility as compared to static metasurfaces, the use of the DMD, laser, and projection optics prevents a highly compact integration, which would allow for dynamic tuning of a small metasurface device in operandi. To overcome this limitation, in a next step, we further harness the multiresponsivity of the LC. Specifically, we show that the spatially engineered domains of different in-plane alignment directions, and thereby the spatial variation of the metasurface response, can be temporarily erased by heating the system over the critical temperature of the E7 LC (*T*_c_ = 58 °C), where it undergoes a phase change from nematic to isotropic. At the device level, this may allow switching functionalities, such as the display of images or diffractive elements, that rely on the spatially variable response “on” and “off” by the simple application of a global stimulus–here heat–which can be realized in a highly compact fashion. Note that the same effect may, in principle, be achieved with the application of a voltage across the LC cell. In our experiment, the LC cell is gradually heated from 20 to 60 °C during a 3 min interval. Corresponding time-resolved transmission measurements recorded inside (*T*_⊥_) and outside (*T*_∥_) of the DMD-exposed region are shown in Figure 4a for 694 nm wavelength and *y*-polarized incident light. The transmission remained approximately constant for a duration of ∼150 s before an abrupt change in the transmittance was observed. This change is attributed to the phase transition of the LC from the nematic to the isotropic phase. The phase transition causes a change of the refractive index of the LC to *n* = 1.57^31^ and thus, a shift of the ED and MD resonances. Transmission spectra of the LC-integrated metasurface for the isotropic phase are included in the Supporting Information. Once in the isotropic state, as expected for an isotropic embedding medium, no difference is observed between *T*_∥_ and *T*_⊥_. Next, we allowed the LC cell to cool back to room temperature. Corresponding time-resolved transmission measurements are shown in Figure 4b. The phase transition from the isotropic back to the nematic phase occurred after ∼70 s, after which the transmission reverted back to the initial values observed before heating. Note that the strongest switching dynamics occur within a second time scale, providing the possibility for fast switching in set point-controlled devices. The shift of the ED and MD resonances due to the thermo-optical effect of the silicon is on the order of few nanometers for the considered temperature range and can be neglected.^60^ To provide direct evidence of the erasure and reappearance of the spatially engineered domains of different in-plane alignment directions defined by DMD exposure, we furthermore recorded the spatially resolved transmission of the LC-integrated metasurface at 694 nm before heating, at *T* > *T*_c_, and after cooling down. These results are presented in Figure 4c,d,e, respectively. Importantly, after cooling, the transmission recovers back to the original value before heating, indicating that the LC photoalignment mechanism is not affected by the applied temperatures.

## Conclusions and Outlook

We have experimentally demonstrated the use of polarized blue light as an external stimulus for the active tuning of LC-integrated Mie-resonant metasurfaces in a spatially controlled fashion. To this end, we used spatially structured polarized light in combination with photoalignment layers to spatially engineer domains of different in-plane alignment directions within the LC metasurface cell. We have employed this approach for spatially controlled switching of the metasurface transmittance, mediated by the spectral shift of its supported Mie resonances upon the induced change in the dielectric environment of its constituent meta-atoms. Importantly, the switching is reversible and stable over several cycles. Moreover, making use of the multiresponsive nature of the LCs, by heating the LC cell above the critical temperature of the LC, we erased the previously defined patterns. Upon cooling to below the critical temperature, the patterns were fully restored. Depending on the application, the temporary pattern erasure by heating could be replaced by the application of a voltage along the *z*-direction of the LC cell. Furthermore, the time for the reorientation of the LC molecules could be reduced by increasing the intensity of the incident light to 450 nm during the exposure. As such, our approach can facilitate the development of image displays and various reconfigurable and switchable optical components, including arbitrarily shaped apertures or pinholes. Further enhancement of the patterning resolution may open a pathway toward reconfigurable and switchable diffractive optical components, such as gratings, Fresnel zone plates, or holographic amplitude masks. Independent of spatial resolution, owing to the resonant response of the metasurfaces, the mentioned reconfigurable components have the potential to modulate a specific frequency, polarization, or incidence angle of the incident light only, while the sample would appear largely homogeneous off resonance, thereby going far beyond the case of simple reconfigurable or tunable amplitude masks. More generally, combining the vast opportunities for light manipulation by tailored resonant metasurfaces in combination with our demonstrated approach allows for tailoring a complex multiresponsive spatiospectral response of the overall system, thereby accessing new functionalities not offered by conventional LC-cell-based SLMs.

## Supporting Information Available

Near-field profiles of the LC integrated metasurface; simulated and measured transmission spectra of LC metasurface cell in isotropic LC state; detailed description of the construction process of the LC cell; description of the optical setups used for spatially controlled blue light exposure and for spatially resolved transmission measurements; demonstration of a wavelength-dependent contrast reversal of exposed and prealigned regions; and comparison for nominally identical system configurations of spectra for multiple switching cycles (PDF)
