# Supplementary material for: Spatially Controlled All-Optical Switching of Liquid-Crystal-Empowered Metasurfaces
Source: ACS Photonics. 2025 Jan 21;12(2):963–70. doi: 10.1021/acsphotonics.4c02029 (PMC11844236; doi:10.1021/acsphotonics.4c02029)
Supplement: Supplementary file 1 — ph4c02029_si_001.pdf [file ph4c02029_si_001.pdf]

Spatially-Controlled All-Optical Switching of Liquid-Crystal Empowered Metasurfaces: Supporting Information

Maximilian Beddoe,[*,†,‡] Sarah L. Walden,[†,‡,¶] Slobodan Miljevic,[†,‡] Dmitry Pidgayko,[†,‡] Chengjun Zou,[†,‡,§] Alexander E. Minovich,[†,‡] Angela Barreda,[†,‡,∥] Thomas Pertsch,[‡,⊥] and Isabelle Staude[*,†,‡]

†*Institute of Solid State Physics, Friedrich Schiller University Jena, 07743 Jena, Germany*

‡*Institute of Applied Physics, Abbe Center of Photonics, Friedrich Schiller University Jena, 07745 Jena, Germany.*

¶*School of Environment and Science, Griffith University, Nathan 4111, Australia.*

§*Institute of Microelectronics, Chinese Academy of Sciences, Beitucheng west road 3, Beijing, China*

∥*Group of Displays and Photonics Applications, Carlos III University of Madrid, 28911 Madrid, Spain*

⊥*Fraunhofer Institute for Applied Optics and Precision Engineering, 07745 Jena, Germany*

E-mail: maximilian.edmund.beddoe@uni-jena.de; isabelle.staude@uni-jena.de

Number of pages: 9

Number of figures: 6

S1. Near-field profile of the metasurface embedded in LC

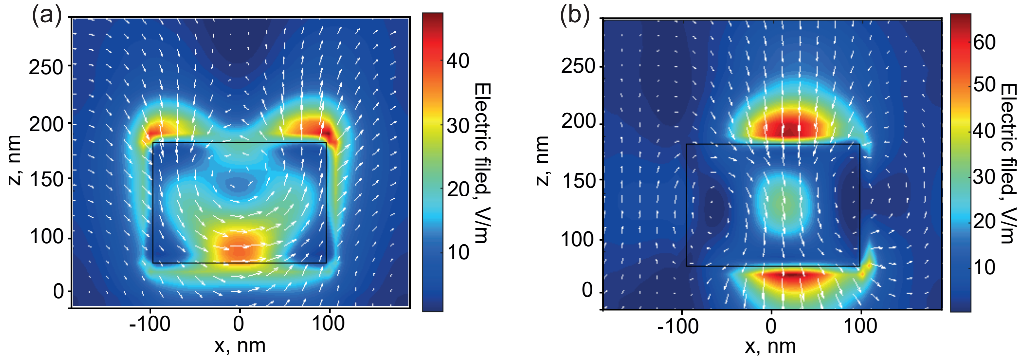

Figure S1: Near-field profiles in the unit cell of the metasurface (x-z plane through the centre of the nanocylinder) for incident light polarized perpendicular to orientation of LCs ($E_{inc} \perp$ LC) at (a) 710 nm and (b) 670 nm. The white arrows represent the polarization vectors of the electric field E.

Figure S1 shows the near-field plots of the resonances in the pre-aligned region ($E_{inc} \perp$ LC). The resonance at 670 nm (Figure S1 (a)) corresponds to the collective ED and MD resonance,[1] which are very close.

The resonance at 670 nm (Figure S1 (b)) is an out-of-plane electric dipole, which cannot be excited by normal incidence radiation (dark modes).[2] In fact, this dark mode can be accessed by normal incidence due to the symmetry break induced by the anisotropic LC refractive index.[2]

S2. Transmission of LC cell in isotropic state

Figure S2 shows the comparison of the simulated and measured transmittance spectrum for the liquid crystal in the isotropic state with a refractive index of 1.5753.[3,4] The molecular disorder removes the polarization dependence of the transmission.

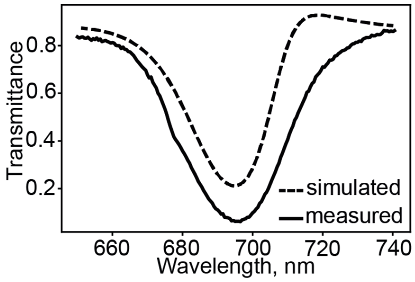

Figure S2: Numerically calculated and measured transmittance spectra for LC in isotropic phase.

S3. Detailed construction process of LC cell

The cell was assembled starting from a SiO_2 glass cover plate and the metasurface sample, both having dimensions of 20 mm $\times$ 30 mm. As a first step, they were cleaned with acetone, isopropanol and de-ionized water. Afterwards, they were placed into an oxygen plasma cleaner for 5 minutes. This step is required to remove any organic contamination. Subsequently, a photoalignment layer was prepared by dissolving AtA-2 (in powder form) in dimethylformamide (DMF) at a concentration of 0.25 weight %. The solution was then filtered using a 0.2 μm filter. Next, the AtA-2 solution was spin-coated on the surface of the cleaned glass plate and the metasurface sample. To this end, both were heated up using a hot plate for 2 minutes to 80 °C and then quickly placed on the spin-coater. Spin coating was performed at a speed of 800 rpm for 5 s, followed by 3000 rpm for 40 s and finished with 800 rpm for 5 s. Next, the spin-coated samples were baked on a hot plate for 5 minutes at 140 °C. This process resulted in a photoalignment layer of approximately 15 nm thickness.[2] Afterwards, the glass plate and the metasurface sample were illuminated with x-polarized blue light (100 W blue LED, 450-455 nm) for 90 s. To form the actual cell, the glass plate and the metasurface sample were pressed together using a metallic sample clip, which has a 20 mm $\times$ 20 mm window. In order to determine the cell thickness, we recorded the transmittance spectra of the empty cell and fitted them with a Fabry-Perot model.[5] The determined thickness was used to estimate the amount of LC needed to fully infiltrate the cell. The LC

was injected with a pipette at the edge of the assembled cell. The capillary effect spreads the LCs evenly throughout. Finally, the assembled and infiltrated cell was placed in the constructed DMD setup (Figure S7). The sample was exposed with y-polarized light with a wavelength of 457 nm at a power of 41 mW. The sample was heated during the exposure to bring the LC in its isotropic state, thereby avoiding effects of polarization rotation as common for twisted LC cells. The necessary temperature increase above the critical temperature of the LC was achieved using a 15 Ω heating resistor attached at the sample backside and a PT100 RTD temperature sensor feedback system controlled with a temperature controller (LDT-5980, Newport).

S4. Experimental setup for spatially-controlled exposure

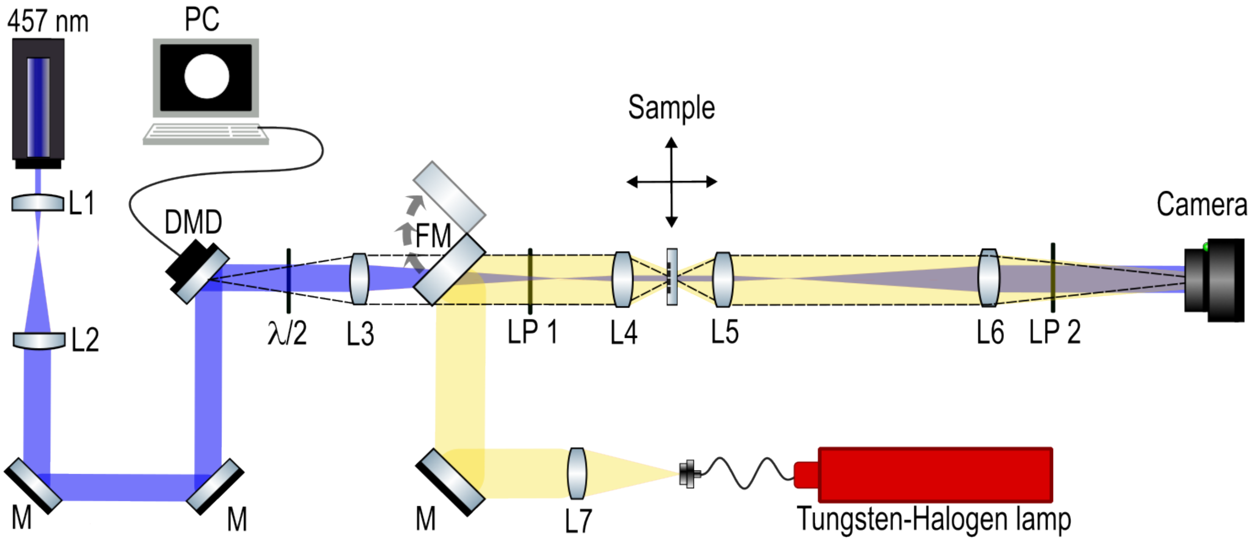

Figure S3: Sketch of the optical setup used for spatially-controlled blue-light exposure of the LC metasurface cell

Spatially-controlled blue-light exposure of the LC metasurface cell was performed using a custom-built optical setup. A sketch of the setup is shown in Fig. S3. A digital mirror

device (DMD, DLP3310 0.33 1080p, Texas Instruments) was illuminated with a cw laser beam (Photontec 457nm DPSS) expanded with lenses L1 with focal lengths of $f_{L1} = 20$ mm and $f_{L2} = 50$ mm. A de-magnified image of the DMD output was then projected onto the LC metasurface cell using lenses L3 ($f_{L3} = 200$ mm) and L4 ($f_{L4} = 30$ mm). To select the desired polarization with maximum power efficiency, a halfwave plate ($\lambda/2$) and a linear polarizer (LP 1) were employed. Finally, lenses L5 ($f_{L5} = 25$ mm) and L6 ($f_{L6} = 100$ mm) were used to image the sample onto a CMOS camera, where a linear polarizer LP2 acted as an analyzer. To allow for incoherent white-light imaging of the sample in the same setup, the output of a stabilized tungsten-halogen light source (Thorlabs SLS201L) was collimated with the help of the lens L7 ($f_{L7} = 12$ mm) and introduced into the setup via the flip mirror FM, blocking the laser light. The black dashed line denotes the optical path relevant for white-light imaging. The screen, which was projected from the DMD onto the sample exhibits an area of 746 μm x 1400 μm.

S5. Spatially resolved transmittance measurements

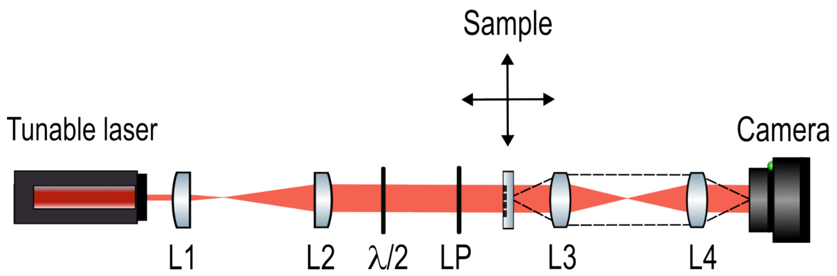

Figure S4: Sketch of the optical setup used for spatially resolved transmittance measurements.

The spatially resolved transmittance of the LC metasurface cell was measured using the custom built setup sketched in Fig. S4. In this setup, a supercontinuum laser (SuperK Extreme, NKT Photonics) was used as a light source. The outcoming wavelength was selected with a RF-based monochromator (SuperK Select). Next, the beam was expanded

by lenses L1 ($f_{L1} = 10\,mm$) and L2 ($f_{L2} = 50\,mm$). The desired incident polarization was set using a halfwave plate ($\lambda/2$) and a linear polarizer (LP). Finally, an image of the sample was focused onto a camera (Zelux 1.6 MP Monochrome CMOS Camera) using lenses L3 ($f_{L3} = 50\,mm$) and L4 ($f_{L4} = 50\,mm$).

S6. Wavelength-dependent contrast reversal of exposed and pre-aligned regions

The resonance shift affected by the reorientation of the LC molecules during the exposure causes a shift of the ED and MD resonance and a contrast in the transmittance at the spectral positions of the resonance in the pre-aligned and the exposed region. This enables to demonstrate a contrast reversal of the exposed region and pre-aligned region by changing the wavelength of the incident light E_{inc}, which is not possible to achieve with the metasurface in the main manuscript. Thus, another metasurface with slightly different geometrical parameters was used for this experiment. The silicon nanodisk exhibits a radius $r = 97\,nm$, a height of $h = 98\,nm$, a SiO_2 pedestal resulting from slight over-etching with a height of $h_e = 81\,nm$ and a period of $a = 380\,nm$. In the other sample, all the geometrical parameters are similar except h_e. This causes a shift of the ED and MD resonances. For $T_\perp$ (Figure S6 (a)), the response is dominated by a broad resonance at 670 nm, originating from the partial overlap of the ED and MD resonance.[1] This also applies for $T_\parallel$. The resonance shifts to 694 nm, but it is not as shallow as for the sample in the main manuscript (Figure S5 (a)). We calculated the difference spectrum $\Delta T = T_\parallel - T_\perp$ (see Figure S5 (a) bottom). The maximum is located at 674 nm and the minimum at 694 nm. To demonstrate the contrast reversal, we exposed other metasurface sample with varied geometrical parameters with a circular-shaped beam of blue light of 450 nm. Next, the spatially-resolved transmittance of the exposed region was recorded at 674 nm (Line i) in Figure S5 a) and Figure S5 (b)) and 694 nm (Line ii) in Figure S5 a)Figure S5 (c)). At 674 nm, the expected decrease of the transmittance in the exposed

region was observed, whereas the transmittance increased in the exposed region at 694 nm. These results show that the contrast between the exposed region and the pre-aligned region can be reversed by changing the wavelength.

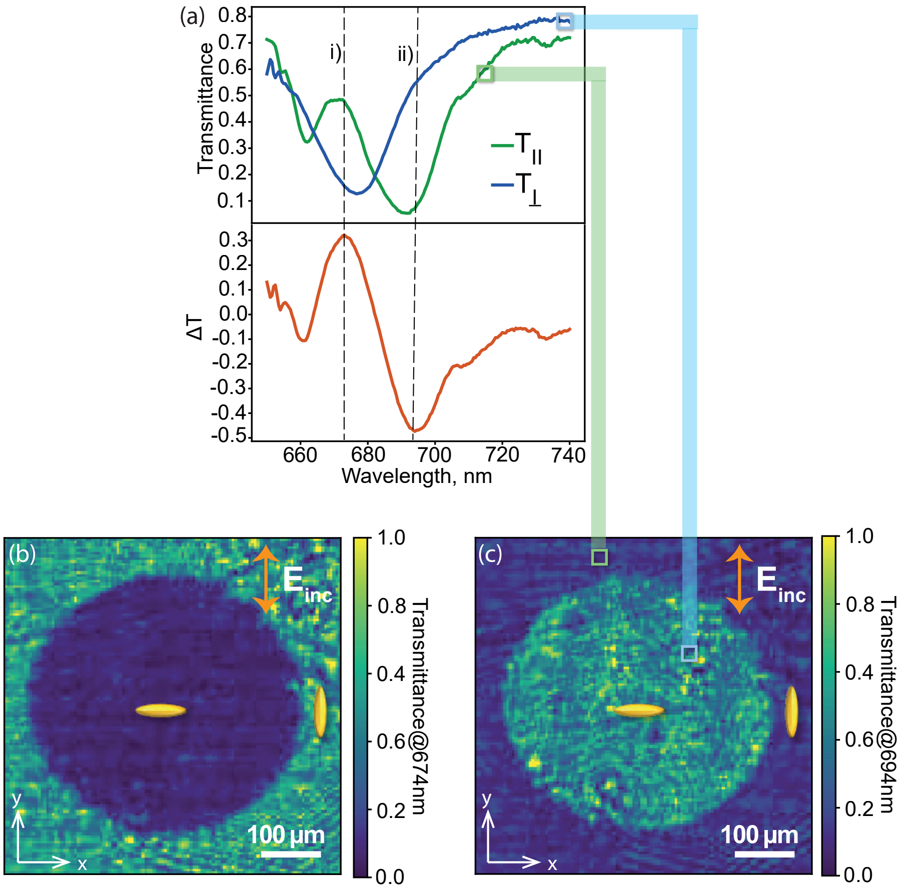

Figure S5: (a) Experimentally measured transmittance spectra of the other metasurface sample with varied geometrical parameters $T_{\parallel}$ and $T_{\perp}$ for y-polarized incident light and y- and x-oriented LCs, respectively and transmittance difference $\Delta T = T_{\parallel} - T_{\perp}$. Spatially-resolved transmittance at (b) 674 nm and (c) 694 nm.

S7. Comparison of spectra for multiple switching cycles

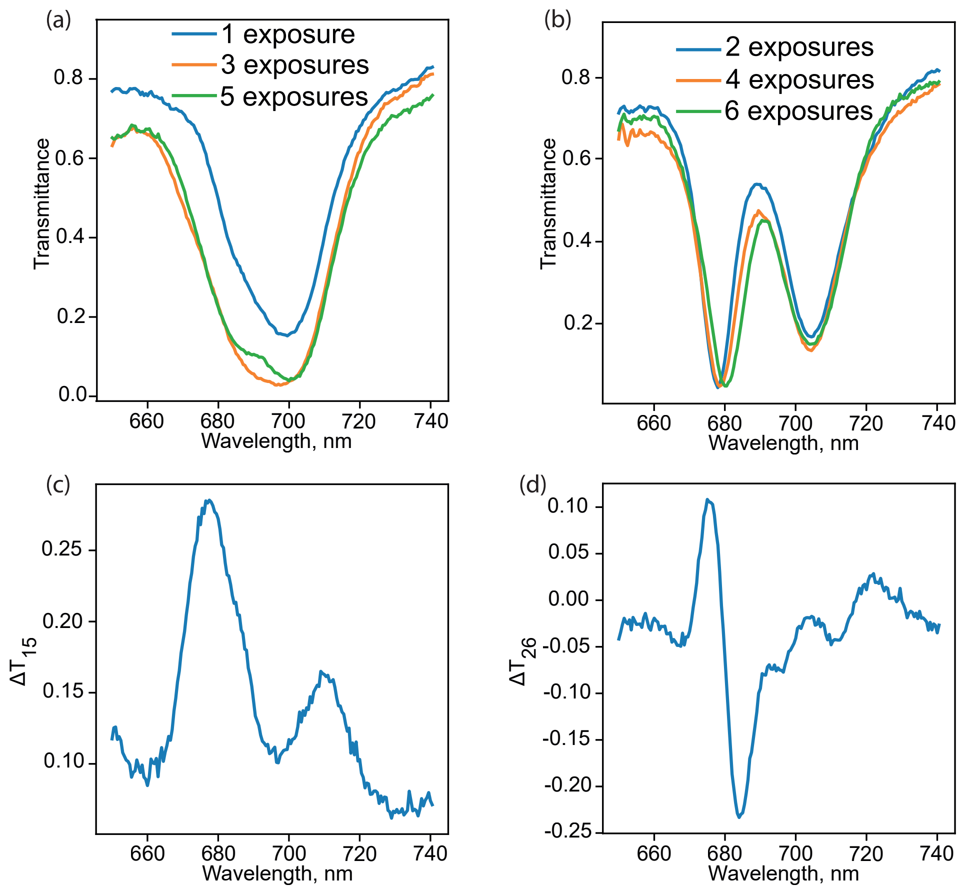

Figure S6: Transmittance spectra for multiple switching. Plot of all (a) odd and (b) even exposures. (c) Difference $\Delta T_{15} = T_1 - T_5$ with T_1 being the transmittance for 1 exposure and T_5 being the transmittance for 5 exposures. (d) Difference $\Delta T_{26} = T_2 - T_6$ with T_2 being the transmittance for 2 exposures and T_6 being the transmittance for 6 exposures.

References

1. Decker, M.; Staude, I.; Falkner, M.; Dominguez, J.; Neshev, D. N.; Brener, I.; Pertsch, T.; Kivshar, Y. S. High-Efficiency Dielectric Huygens' Surfaces. *Advanced Optical Materials* **2015**, *3*, 813–820.

2. Zou, C.; Komar, A.; Fasold, S.; Bohn, J.; Muravsky, A. A.; Murauski, A. A.; Pertsch, T.; Neshev, D. N.; Staude, I. Electrically Tunable Transparent Displays for Visible Light Based on Dielectric Metasurfaces. *ACS Photonics* **2019**, *6*, 1533–1540.

3. Zou, C.; Amaya, C.; Fasold, S.; Muravsky, A. A.; Murauski, A. A.; Pertsch, T.; Staude, I. Multiresponsive Dielectric Metasurfaces. *ACS Photonics* **2021**, *8*, 1775–1783.

4. Li, J.; Wu, S.; Brugioni, S.; Meucci, R.; Faetti, S. Infrared refractive indices of liquid crystals. *Journal of Applied Physics* **2005**, *97*, 073501.

5. Hernandez, G. J. *Fabry-Perot Interferometers*; Cambridge University Press, 1986.
